# The Small GTPase Cdc42 Is a Major Regulator of Neutrophil Effector Functions

**DOI:** 10.3389/fimmu.2020.01197

**Published:** 2020-06-12

**Authors:** Heidi Tackenberg, Sonja Möller, Marie-Dominique Filippi, Tamás Laskay

**Affiliations:** ^1^Department of Infectious Diseases and Microbiology, University of Lübeck, Lübeck, Germany; ^2^Division of Experimental Hematology and Cancer Biology, Cincinnati Children's Hospital Medical Center, Cincinnati, OH, United States

**Keywords:** small GTPases, Cdc42, neutrophil granulocytes, ROS, degranulation, migration, pathogen killing

## Abstract

Neutrophil granulocytes are key components of the innate immune system. As the first responders to inflammatory cues, they rapidly migrate toward the site of infection or inflammation and fulfill diverse effector functions. Since these effector functions can be both beneficial and harmful to the host and surrounding tissue, they require a strict control. The small GTPase Cdc42 is known to regulate neutrophil locomotion by controlling cytoskeleton rearrangement in murine neutrophils. However, the role of Cdc42 in other neutrophil functions in human neutrophils is still poorly understood. Here we demonstrate that in primary human neutrophils, Cdc42 controls directed and random migration, activation, and degranulation as well as the formation of reactive oxygen species, in a stimulus dependent manner. In addition, we show that Cdc42 regulates pathogen killing efficiency, both in murine and human neutrophils. Cdc42 regulation of neutrophil functions is linked to differential regulation of Akt, p38, and p42/44. Our data, therefore, suggests a mechanistic role for Cdc42 activity in primary human neutrophil biology, and identify Cdc42 activity as a target to modulate neutrophil effector mechanisms and killing efficacy.

## Introduction

Neutrophil granulocytes, as the first line of defense of the innate immune system are rapidly recruited to and activated at sites of infection or inflammation (1). Circulating neutrophil granulocytes leave the bloodstream upon receiving inflammatory cues and migrate toward the site of infection. Once at the site of infection, neutrophils exert multiple antimicrobial effector mechanisms, among others formation and release of reactive oxygen species (ROS), degranulation, and phagocytosis. All these mechanisms are aiming to efficiently kill pathogens, but can be equally toxic to the host (2). Neutrophil granulocytes are known to play a key role in several autoimmune diseases like systemic lupus erythematosus (SLE), rheumatoid arthritis (RA), or psoriasis (3). It is understood that the sheer presence of neutrophils for example at the joints or the skin, contributes to inflammatory processes. These clinical findings highlight the importance to understand how neutrophil functions are regulated. Small GTPases of the Rho-subfamily are known to control neutrophil recruitment to sites of infection, by regulating cytoskeleton rearrangements. Rho GTPases are molecular switches that cycle between the active GTP-bound and the inactive GDP-bound state (4). This cycling is facilitated by GTP exchange factors (GEFs) and GTPase activating proteins (GAPs). In resting cells, sGPTases exist in their inactive state. Upon stimulation with growth factors, cytokines or chemokines, different Rho-specific guanine nucleotide exchange factors (RhoGEFs) are activated, depending on the triggered receptors. Additonally to the GDP/GTP exchange, Rho GTPases are regulated by changes in their subcellular location and require docking onto the cell membrane to exert their cellular functions (4). Once activated and translocated to their specific subcellular location, Rho GTPases can interact with several effectors, and therefore are capable of engaging specific signaling cascades (5).

The Rho GTPase Cdc42 is a known regulator of actin and tubulin organization, cell-cell and cell-extracellular matrix interactions as well as cell polarity, in different cell types (4, 6, 7). As such, Cdc42 activation is important for cell migration upon chemoattractant stimulation (5). The role of Cdc42 in neutrophil migration has mostly been characterized in HL-60 cells or murine neutrophils. However, the role of Cdc42 in primary human neutrophil migration and, more importantly, in their effector mechanisms is still poorly understood. To study the influence of Cdc42 on various neutrophil effector mechanisms, the Cdc42 activity-**s**pecific inhibitor (casin) was used. Casin is a Pir1 analog, and confers the most active Cdc42 binding activity without binding to related RhoGTPases (7–9). Casin was shown to specifically bind to Cdc42, and to competitively interfere with its guanine nucleotide exchange (GEF) activity, therefore, suppressing Cdc42-GTP formation (8). Inhibition of Cdc42 with 10 μM casin was shown to be most efficient to reduce cell adhesion and migration, without affecting Rac1-GTP formation. Casin is superior to other Cdc42 inhibitors, such as ML141 or AZA1, since these inhibitors also target other Rho GTPases such as Rac1 or RhoA (7–9). Therefore, no other highly selective Cdc42 inhibitor is available. The present work aimed to clarify the role of Cdc42 in migration, antimicrobial effector mechanisms and bacterial killing in primary human neutrophils by using *in vitro* studies.

## Methods

### Neutrophil Isolation

Primary human neutrophils were isolated from peripheral blood of healthy volunteers by Histopaque and Percoll gradient centrifugation as described elsewhere (10). The blood collection was conducted with the understanding and consent of each participant and was approved by the ethical committee of the Medical Faculty of the University of Lübeck (18-187). Blood was layered on top of a density gradient consisting of Histopaque 1119 and 1077 and centrifuged 5 min, 54 × g and further 25 min 216 × g at room temperature (RT). To further increase the purity of the granulocyte sample, and remove most of the remaining erythrocytes in the granulocyte fraction, a second gradient centrifugation was performed. Granulocytes were collected and added on top of a Percoll gradient consisting of 65–85% Percoll. Following centrifugation for 30 min, 863 × g, granulocytes were collected. The cell preparations contained >99% granulocytes as determined by morphological examination of Giemsa-stained cytocentrifuged slides (Shandon, Pittsburgh, PA). PMNs were cultured in complete medium; RPMI 1640 medium supplemented with 10 mM HEPES, 10% heat inactivated fetal bovine serum (Sigma-Aldrich, Steinheim, Germany), 4 mM L-glutamine (Biochrom, Berlin, Germany).

### Neutrophil Migration Assay *in vitro*

Neutrophil migration was evaluated in triplicate using 3 μm transwell inserts in a 24-well plate (Costar, Kennebunk ME, USA). In the chemotaxis assay, fMLP (1 μM), C5a (10 nM), LPS (100 ng/ml) (all from Sigma-Aldrich, Steinheim, Germany) were added in the lower chamber. In the chemokinesis assay, stimulants were added in the upper chamber with the cells and in the lower chamber. The cells (6 × 10^5^) were pre-incubated with or without 10 μM of the Cdc42 inhibitor casin for 30 min at 37°C and subsequently diluted in 100 μl complete medium with or without fMLP, LPS or C5a. The migration toward the stimulators was allowed for 30 min at 37°C. The number of migrated cells was evaluated by analyzing the glucuronidase activity of lysed cells after migration.

### Neutrophil Activation and Degranulation

To determine neutrophil activation (CD62L-shedding), CD11b, CD18, and CD63 expression upon stimulation, neutrophils (5 × 10^5^) were pre-incubated with or without the Cdc42 inhibitor casin (10 μM) for 30 min at 37°C, followed by stimulation with or without 1 μM fMLP, 10 nM C5a, or 100 ng/ml LPS for 1 h at 37°C. The cells were washed with FACS buffer (PBS supplemented with 1% BSA, 1% human serum) at 4°C, and stained with APC-labeled antibody to CD63 (Merck Millipore, Billerica, Massachusetts, USA), FITC-labeled antibody to CD62L, phycoerythrin (PE)-labeled antibody to CD11b or APC-labeled antibody to CD18 (all from BD, Franklin Lakes, NJ, USA) at 4°C for 20 min. Integrin and L-selectin expression was analyzed by flow cytometry using FACSCanto II (BD, Franklin Lakes, NJ, USA). Fluorescence intensity is reported as normalized mean fluorescence intensity ± SD.

### Production of Reactive Oxygen Species (ROS)

#### Luminol-Amplified Chemiluminescence Assay

The sum of intra- and extracellular ROS was measured using a luminol-amplified chemiluminescence assay. Myeloperoxidase-derived metabolites cause the excitation of luminol (5- amino-2,3- dihydro-1,4-phthalazindione) (11) and they are released from azurophil granules during neutrophil activation and subsequent degranulation. This fact makes the measurement of intra- and extracellular ROS with luminol possible. Following isolation, neutrophils (4 x 10^5^) were seeded into a flat bottom 96-well white-lumitrac plate (Greiner Bio-One, Frickenhausen, Germany) and pre-incubated with or without 10 μM casin for 30 min at 37°C. Subsequently, 0.06 mM luminol (Sigma Aldrich, Missouri, USA) was added and the cells were stimulated with 1 μM fMLP, 10 nM C5a, or 100 ng/ml LPS. Untreated and unstimulated neutrophils served as control. The chemiluminescence, resulting from ROS formation was analyzed immediately using an infinite 200 reader and the Tecan i-control 1.7 Software (Tecan, Crailsheim, Germany). ROS release was monitored every 2 min for a period of 2 h at 37°C.

#### Detection of Intracellular ROS

To specifically determine the formation of intracellular ROS by flow cytometry, the substrate DHR123 (Invitrogen, Eugene, USA) was used. This substrate diffuses into the cells and is oxidized by ROS to the fluorescent Rhodamine123. Freshly isolated neutrophils (5 × 10^5^) were incubated with or without 10 μM casin for 30 min at 37°C. Subsequently, 2 μM DHR123 was added, and the cells were stimulated with 1 μM fMLP, 10 nM C5a, or 100 ng/ml LPS for 5 min at 37°C. The reaction was stopped on ice, and the fluorescence intensity was analyzed immediately by flow cytometry using FACSCanto II.

### Assessment of Bacterial Phagocyosis of Neutrophil Granulocytes

To determine if Cdc42 inhibition or deficiency affects the phagocytosis of *E. coli* or *S. aureus*, the number of ingested bacteria per 100 neutrophil granulocytes was counted. Freshly isolated primary human or bone marrow derived murine neutrophils (9 × 10^5^) were incubated with or without 10 μM casin for 30 min at 37°C. Following centrifugation, cells were re-suspended in HBSS supplemented with 20 mM HEPES (Thermo Fisher, PAA, Pasching, Austria) and incubated together with *E. coli* or *S. aureus* (9 × 10^6^) for 30 min at 37°C while shaking with 216 × g. Subsequently, 100,000 neutrophils were cytocentrifuged for 5 min, 28 × g at RT, followed by Diff Quick staining. The number of ingested bacteria within 100 neutrophil granulocytes was counted using a bright field microscope.

### Assessment of Bacterial Killing by Neutrophil Granulocytes

To determine if Cdc42 inhibition affects the killing of *E. coli* or *S. aureus*, an *in vitro* killing assay was performed. Freshly isolated primary human or bone marrow derived murine neutrophils (9 × 10^5^) were incubated with or without 10 μM casin for 30 min at 37°C. Following centrifugation, cells were re-suspended in HBSS supplemented with 20 mM HEPES (Thermo Fisher, PAA, Pasching, Austria) and incubated together with *E. coli* or *S. aureus* (9 × 10^6^) for 30 min at 37°C while shaking with 216 × g. Subsequently, neutrophils were lysed using lysis medium (HBSS supplemented with 20 mM HEPES, 1 mg/ml Saponin) for 10 min at RT. A 1:2 bacterial dilution series (starting concentration of 1 × 10^9^ bacteria/ml) over 12 wells in LB-medium (Difco LB broth, Lennox, BD) was prepared in a 96-well plate. Samples were prepared in a 1:5 dilution with LB medium into the same plate. The bacterial re-growth was assessed by measuring the optical density (OD) at 650 nm over a period of 15 hrs at 37°C using an infinite 200 reader and Tecan i-control 1.7 Software (Tecan). Bacterial survival was calculated by interpolating the OD of the serial dilution standard curve and the samples and performing a non-linear regression fit analysis.

### MAPK Phosphorylation Analysis

Neutrophils (5 × 10^6^) were first incubated with or without the Cdc42 inhibitor casin (10 μM) for 30 min at 37°C. Cells were then stimulated with 1 μM fMLP for 10 min at 37°C or 100 ng/ml LPS for 30 min at 37°C. Subsequently, cells were lysed with TCA (Sigma Aldrich, Missouri, USA) followed by centrifugation and washing with aceton. Cell lysates were analyzed for phospho-Akt, phospho-p42/44 and phospho-p38 followed by β-actin using western blot analysis. The amount of phosphorylated protein was normalized to β-actin.

### Mice

Murine neutrophils were isolated from bone marrow of Cdc42^fl/fl^ and Cdc42^Δ/Δ^ mice. The use of conventional Cdc42 gene-targeted mice is not possible, since these mice die at the embryonic day 7.5 (12). Therefore, the use of a conditional knock out of Cdc42 in mice was used. The conditional Cdc42 deletion in hematopoietic cells was induced by injecting 3 times Poly (I:C) every other day as described by Yang et al. (12). The efficiency of the Cdc42 deletion was already described by Yang et al., showing that 5 days after injection of 3 doses Poly (I:C), Cdc42 gene sequences and Cdc42 protein became undetectable in bone marrow cells (12). Bone marrow was isolated as described elsewhere (13). The cell suspension was layered on top of a Percoll density gradient consisting of five Percoll concentrations (81, 62, 55, 50, and 45%), followed by centrifugation for 30 min, 550 × g at 10°C. After washing with PBS, cells were re-suspended in 1 x HBSS (supplemented with 0.01% BSA, w/o MgCl_2_, w/o CaCl_2_) and layered on top of Histopaque 1119. Following centrifugation for 20 min, 816 × g at RT, neutrophils were collected and counted with a Hemocytometer (Hemavet 950FS, Miami Lakes, FL, USA).

### Statistical Analysis

Statistical analysis was performed with Graph pad prism 6 Software. Data were analyzed using One way ANOVA or students *t*-test. Data are displayed as mean ± SD. Differences were considered significant if *p* ≤ 0.0001:^****^, *p* ≤ 0.001:^***^, *p* ≤ 0.01: ^**^, *p* ≤ 0.05: ^*^.

## Results

### Inhibition of Cdc42 Activity Is Associated With Changes in Locomotion of Primary Human Neutrophils

Neutrophils express several G-protein coupled receptors (GPCR) on their surface participating in host defense and inflammation (14). Formyl peptide receptors (FPR), classical chemoattractant receptors, and chemokine receptors represent a subfamily of GPCRs. There are three known FPR (FPR1-3) capable of sensing bacterial products and tissue injury by recognizing the release of mitochondrially synthesized proteins or formyl peptides derived from bacteria. Classical chemoattractant receptors respond to leukotriene B4, platelet activating factor (PAF) and also C5a, which are all produced during inflammatory processes (14). A common feature of GPCRs is their 7-trans-membrane structure coupled to G-proteins for signal transduction and their shared ability to induce chemotactic and chemokinetic locomotion of PMNs.

**Figure 1:**
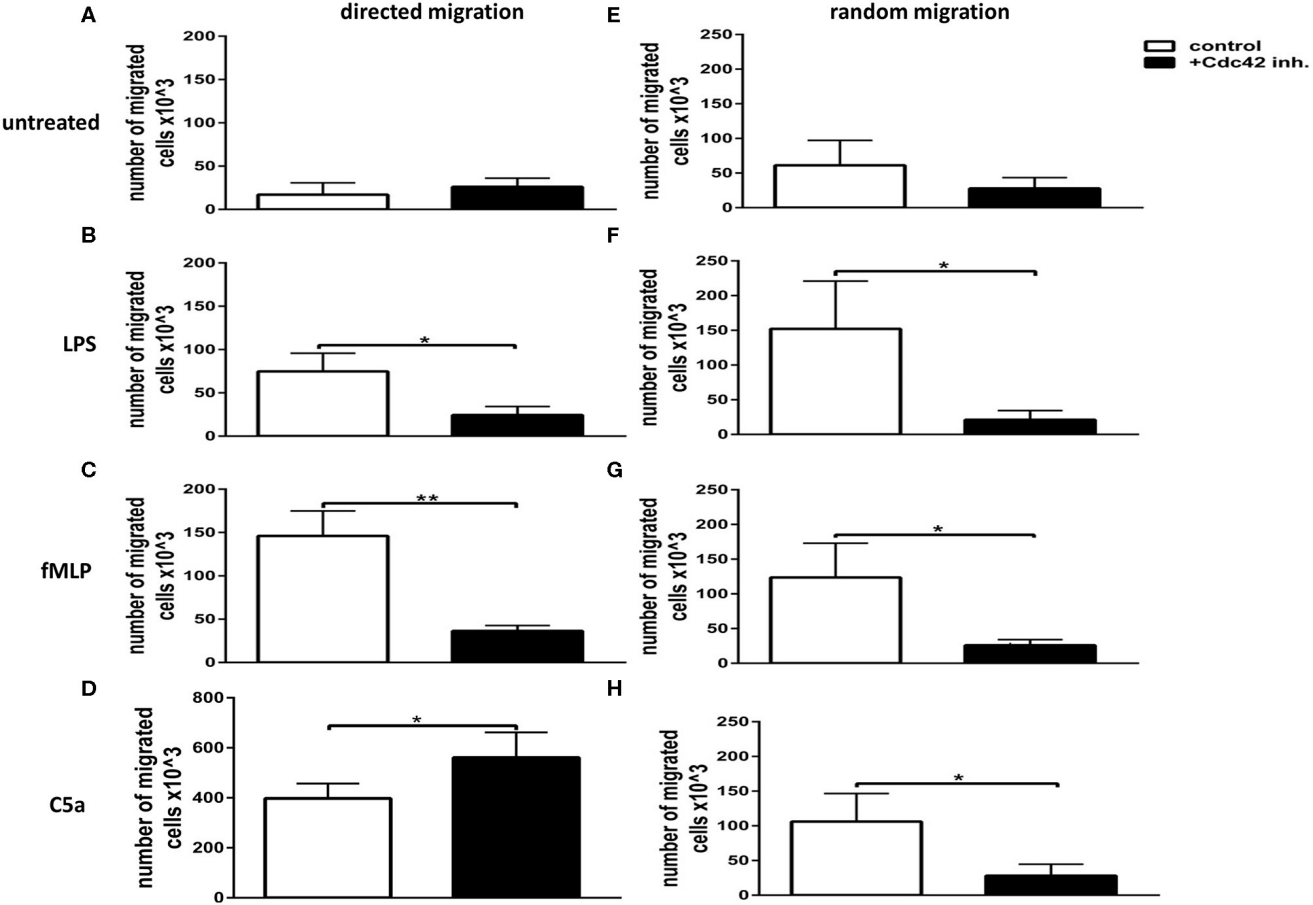
Cdc42 regulates both random movement and directed migration of neutrophils. Neutrophil directed migration **(A–D)** and random movement **(E–H)** were assessed using 3 μm transwell inserts. Migration was evaluated without stimulus **(A,E)** or with 100 ng/ml LPS **(B,F)**, 1 μM fMLP **(C,G)**, or 10 nM C5a **(D,H)**. Random movement was assessed by applying the stimuli both into the lower and upper chamber of the transwell **(E–H)**. Directed migration was assessed by applying the stimuli into the lower chamber **(A–D)**. Neutrophils were left untreated (white bars) or treated with 10 μM Cdc42 inhibitor casin (black bars). Bar graphs represent the number of migrated cells, mean ± SD from three independent experiments assessed by glucuronidase activity analysis. **p ≤ 0.01; *p ≤ 0.05; unpaired, two-tailed students *t*-test.

To address the role of Cdc42 activity in directed and undirected migration, primary human neutrophils were treated with or without the Cdc42 inhibitor casin and allowed to migrate toward different stimuli. To analyze random migration, stimulators were added in the upper and lower chamber of the transwell, while directed migration was assessed by adding the stimulators only in the lower chamber of the transwell. The number of directed migrating cells was significantly decreased in response to fMLP and LPS (Figures 1B,C) whereas it was slightly increased in response to C5a, when Cdc42 was inhibited as compared to untreated cells (Figure 1D). Similarly, the number of randomly moving cells in response to LPS, fMLP, and C5a was significantly reduced when Cdc42 was inhibited as compared to untreated cells (Figures 1F–H).

### Inhibition of Cdc42 Affects the Activation and Secretory Vesicle Degranulation of Neutrophils in a Stimulus Dependent Manner

Neutrophil granulocytes also express receptors aiding cell adhesion, therefore being important for migration to sites of inflammation across epithelial barriers (15). These receptors are called selectins and integrins and interact with a large variety of different carbohydrates containing surface molecules. Integrins are heterodimeric transmembrane glycoproteins present on nearly all mammalian cells. The most important integrins belong to the β2-integrin family, which is formed by the β2-integrin chain (CD18) and a unique chain. Lymphocyte function-associated antigen-1 (LFA1), also known as CD11a/CD18 (α_1_β_2_) is expressed on all circulating leukocytes, while macrophage antigen 1 (Mac1), known as CD11b/CD18 (α_M_β_2_) is primarily expressed on neutrophils, monocytes and macrophages (15). Both integrins, LFA-1 and Mac1, bind to epithelial ICAM-1 and are therefore important during neutrophil adhesion and transendothelial migration.

**Figure 2:**
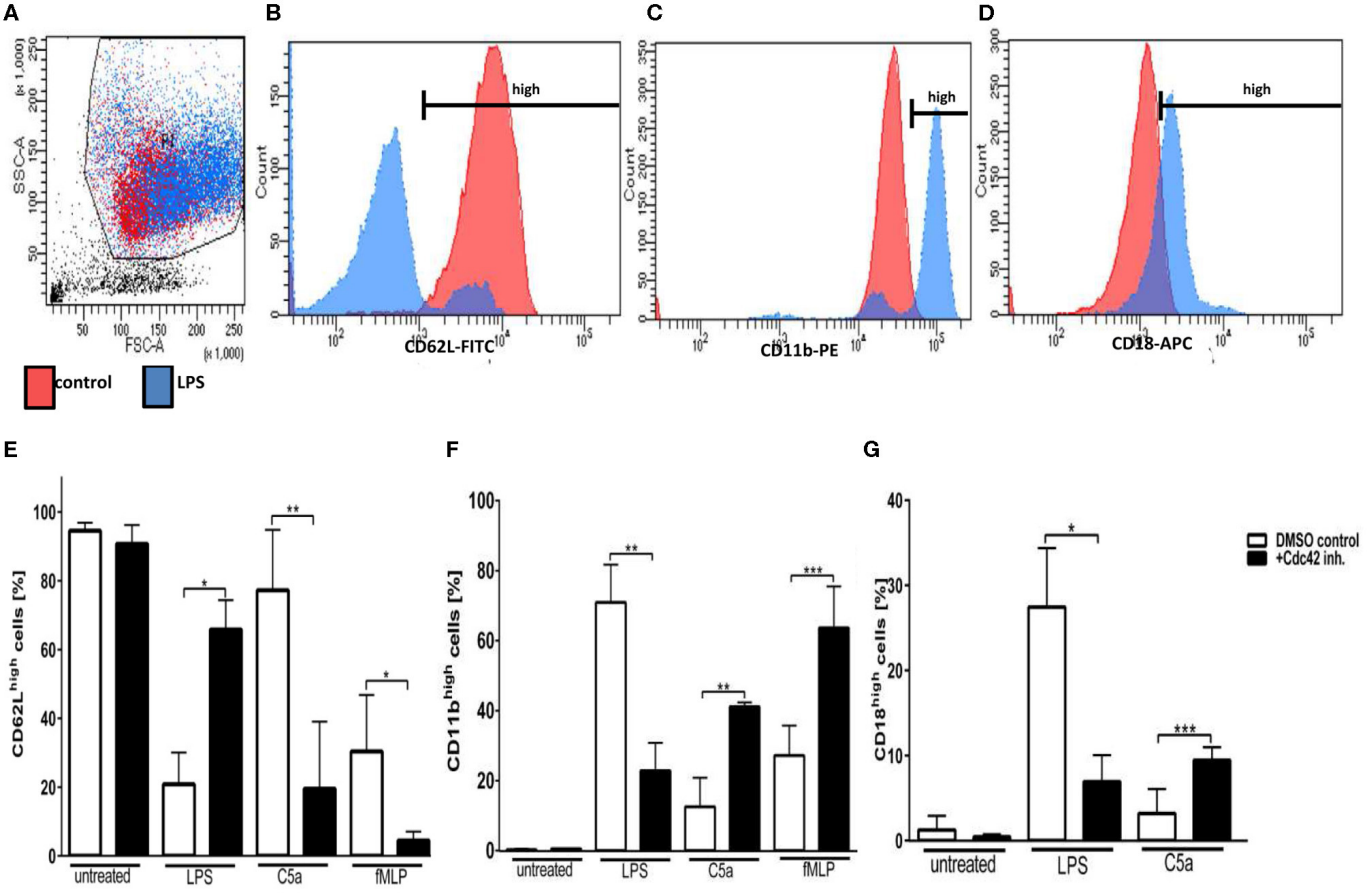
Cdc42 regulates neutrophil activation and secretory vesicle degranulation. Freshly isolated human neutrophils were treated with DMSO (solvent control, white bars) or 10 μM casin (black bars) for 30 min at 37°C. Cells were left untreated or stimulated with 100 ng/ml LPS, 10 nM C5a, or 1 μM fMLP. Neutrophils were stained with antibodies to the activation marker CD62L (FITC), and the degranulation markers CD11b (PE), and CD18 (APC), respectively. Displayed are representative flow cytometry histograms of unstimulated PMNs (red) or cells stimulated with 100 ng/ml LPS (blue). **(A)** Neutrophils were gated according to the size and granularity shown in the sideward- (SSC) and forward-scatter (FSC). **(B–D)** Neutrophils were gated for the activation marker CD62L **(B)** and the degranulation markers CD11b **(C)** and CD18 **(D)**. CD11b^high^, CD62L^high^, and CD18^high^ cells were used for analysis. Bar graphs represent the ratio (% ± SD) from three independent experiments of CD62L^high^ **(E)**, CD11b^high^ **(F)**, and CD18^high^ **(G)** cells assessed by flow cytometry. ***p ≤ 0.001; **p ≤ 0.01; *p ≤ 0.05; unpaired, two-tailed students *t*-test.

To dissect the role of Cdc42 activity in neutrophil activation and degranulation, we next examined the expression of L-selectin (CD62L) and Integrins (CD11b, CD18) in response to different stimuli, according to the gating strategy (Figures 2A–D). L-selectin contributes to physiologic leukocyte rolling and is rapidly shed from the surface of leukocytes upon activation (16). The loss of L-selectin from the surface of leukocytes therefore displays a normal mechanism in response to stimulation. The inhibition of Cdc42 activity did not alter the expression of L-selectin in untreated cells (Figure 2E). In response to LPS, L-selectin expression normally decreases. However, L-selectin shedding by LPS was inhibited by blocking Cdc42 activity (Figure 2E). On the other hand, L-selectin shedding in response to C5a and fMLP was significantly increased by Cdc42 inhibition (Figure 2E). We next examined the expression of the degranulation markers CD11b and CD18. CD11b expression is normally increased in response to inflammatory stimuli. Interestingly, inhibition of Cdc42 activity significantly decreased LPS-induced CD11b expression (Figure 2F). In contrast, Cdc42 inhibited cells showed a significantly increased expression of CD11b and CD18 when stimulated with C5a and fMLP compared to untreated cells (Figures 2F,G).

### Inhibition of Cdc42 Activity Increases the Exocytosis of Primary Granules

**Figure 3:**
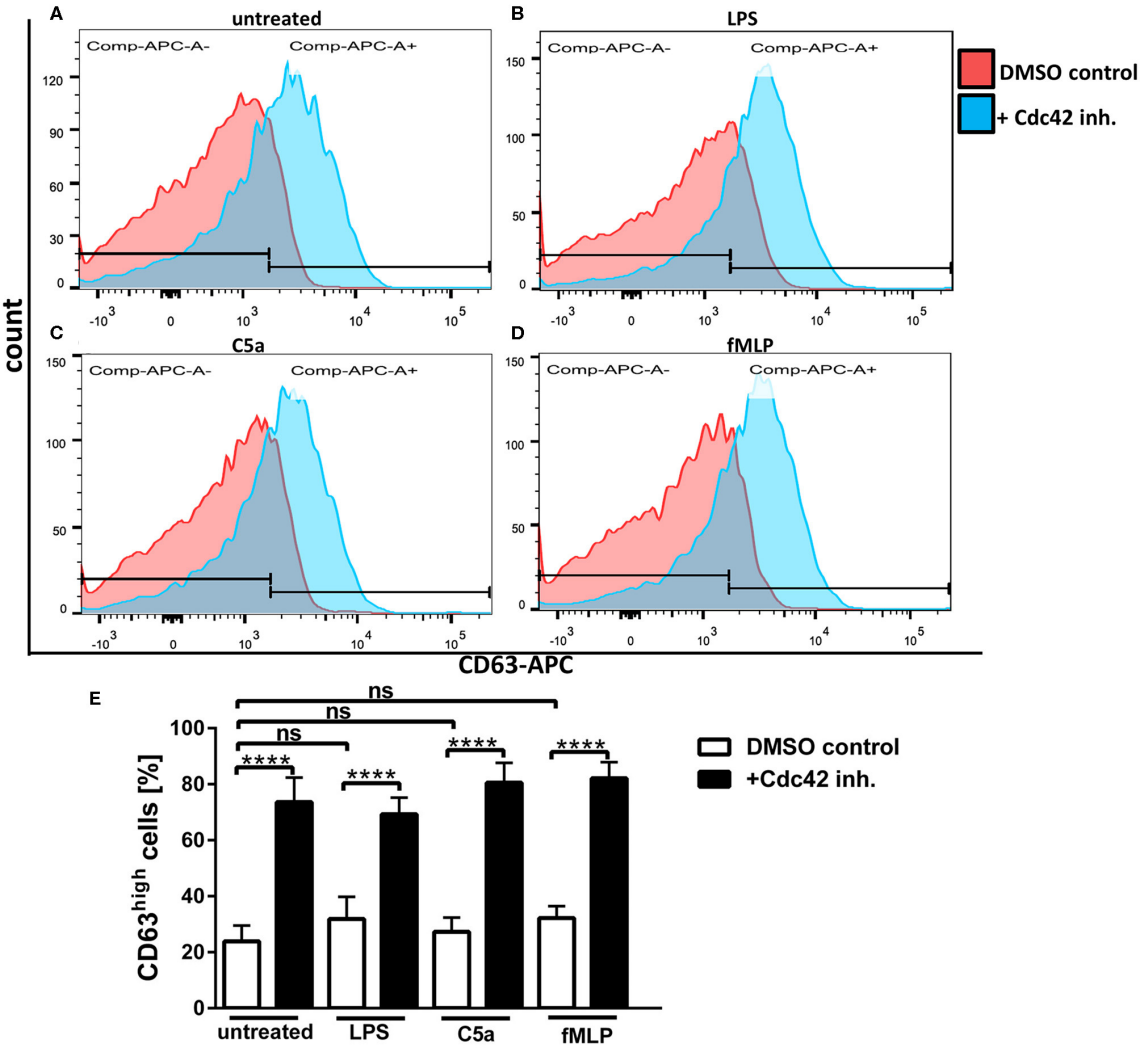
Cdc42 inhibition increases exocytosis of primary granules. Freshly isolated human neutrophils were treated with DMSO (solvent control, white bars) or 10 μM casin (black bars) for 30 min at 37°C. Cells were left untreated or stimulated with 100 ng/ml LPS, 10 nM C5a, or 1 μM fMLP. Neutrophils were stained with an antibody to the degranulation marker CD63 (APC). Displayed are representative flow cytometry histograms of control cells (red) or cells treated with casin (blue) that were either left untreated **(A)**, or treated with LPS **(B)**, C5a **(C)**, or fMLP **(D)**. Neutrophils were gated according to the size and granularity, and subsequently for the marker of primary granule exocytosis CD63. CD63^high^ cells were used for analysis. Bar graphs represent the ratio (% ± SD) from three independent experiments of CD63^high^ **(E)** cells assessed by flow cytometry. ****p ≤ 0.001; ANOVA, Sidak's multiple comparison.

Primary granules, also known as azurophilic granules are the main storage site for the most toxic mediators, including elastase, cathepsin G, and defensins. Moreover, the enzyme myeloperoxidase (MPO) as well as the cell surface protein CD63 are stored in primary granules (17, 18). To assess the influence Cdc42 activity on the exocytosis of primary granules in human neutrophils, we analyzed the surface expression of CD63 in response to different stimulants using flow cytometry. We could show that unstimulated neutrophils show a relatively low expression of CD63 on their surface (Figures 3A,E), which is not significantly increased upon stimulation with LPS, C5a, or fMLP (Figures 3B–E). Inhibition of Cdc42 increased the surface expression of CD63 in unstimulated cells (Figures 3A,E), but also in response to LPS, C5a, and fMLP, compared to the DMSO control cells (Figures 3B–E). Stimulation of Cdc42 inhibited cells did not further increase the expression of CD63, compared to unstimulated cells treated with the Cdc42 inhibitor (Figure 3E).

### Inhibition of Cdc42 Activity Influences the Formation of Reactive Oxygen Species in a Stimulus Dependent Manner

**Figure 4:**
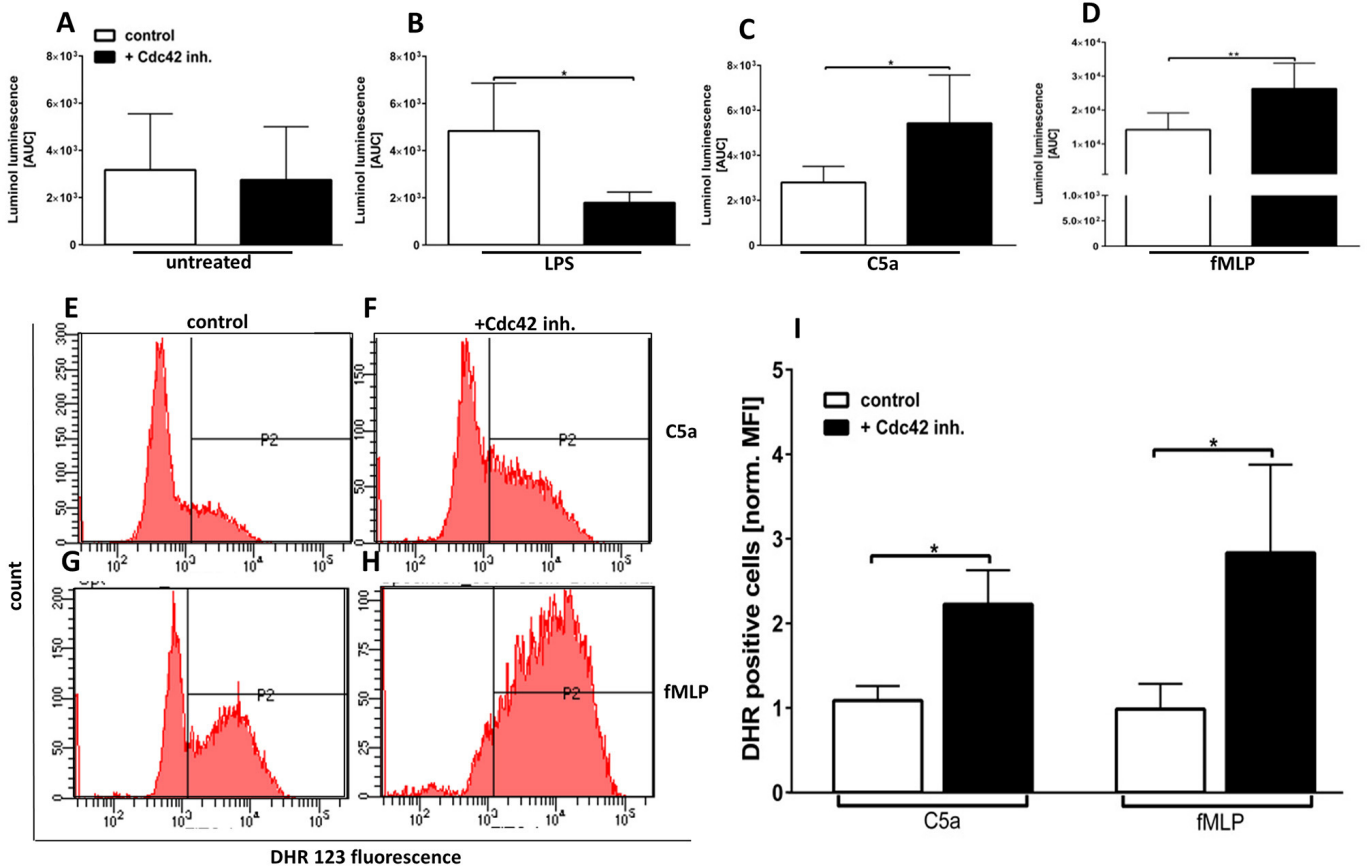
Cdc42 controls the formation of intra-and extracellular ROS in response to LPS, C5a, and fMLP. Primary human neutrophils were incubated with (black bars) or without (white bars) 10 μM casin. Subsequently, luminol was added and the ROS production of unstimulated cells **(A)** or in response to 100 ng/ml LPS **(B)**, 10 nM C5a **(C)**, or 1 μM fMLP **(D)** was measured. Luminol chemiluminescence was detected for 1 h at 37°C and quantified by calculation of the area under the curve (AUC). Bar graphs represent mean ± SD from four independent experiments, **p ≤ 0.01; *p ≤ 0.05; students *t*-test. Neutrophils were incubated with (**F,H,I** black bars) or without (**E,G,I** white bars) 10 μM casin for 30 min at 37°C. Cells were loaded with DHR123, and the samples were stimulated with 10 nM C5a or 1 μM fMLP for 10 min at 37°C. **(E)** Bar graph representing mean fluorescence intensity of DHR123 positive cells normalized to untreated cells, mean ± SD of four independent experiments. *p ≤ 0.05; unpaired, two-tailed students *t*-test.

The GPCR agonists fMLP and C5a are known to induce ROS formation and degranulation but they can also augment responses of PMN to subsequent triggers, a process called priming (14). The NADPH-oxidase (NOX) is one of the major producers of superoxide and its catalytic core is localized at cellular membranes including those of different granules. Since the degranulation is affected by the inhibition of Cdc42 activity, we next sought to elucidate the influence of Cdc42 activity on ROS formation. The luminol-amplified chemiluminescence assay detects both intra- and extracellular ROS since it can be oxidized by several ROS, such as H_2_O_2_, HOCl, HO^•^, or ${\text{O}}_{2}^{•}$ (19, 20). The ROS formation in response to LPS was significantly decreased in Cdc42 inhibited cells as compared to control neutrophils (Figures 4A,B, Supplementary Figure 1). In contrast, in response to C5a and fMLP, ROS formation was significantly increased when Cdc42 is inhibited, compared to untreated controls (Figures 4A,C,D, Supplementary Figure 1).

The intracellular ROS formation was assessed by detecting the fluorescence of oxidized DHR123 to Rhodamine 123 using flow cytometry. There was a significantly higher intracellular ROS production in response to fMLP and C5a in Cdc42 inhibited cells as compared to untreated cells (Figures 4E–I). No changes of intracellular ROS production could be detected in unstimulated and LPS-treated neutrophils (data not shown). This data implicates that Cdc42 activity is required for LPS-induced activation and degranulation as well ROS formation of human neutrophils. Furthermore, the data indicates that Cdc42 is a negative regulator of C5a- and fMLP-induced activation, degranulation, and ROS formation of primary human neutrophils.

### Cdc42 Activity Is Important for *in vitro* Killing of *Staphylococcus aureus* and *Escherichia coli* by Primary Human Neutrophils

**Figure 5:**
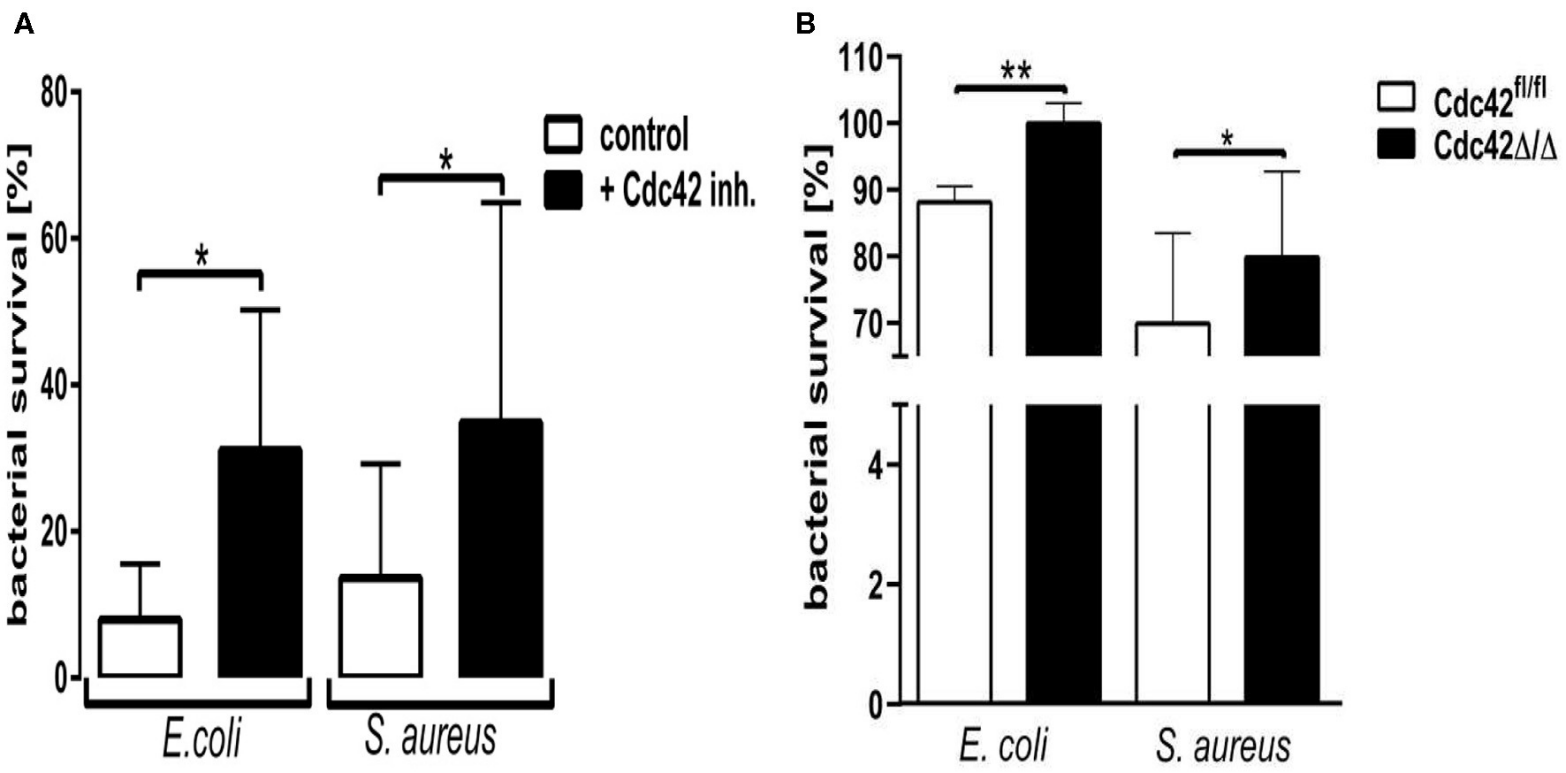
Cdc42 controls the microbial killing efficacy of neutrophil granulocytes. Freshly isolated human neutrophils **(A)**, with (black) or without (white) treatment with 10 μM casin and bone marrow derived murine neutrophils **(B)** from Cdc42fl/fl or Cdc42Δ/Δ mice were incubated with *Escherichia coli* or *Staphylococcus aureus* for 30 min at 37°C and subsequently lysed. The optical density (OD) of bacterial regrowth was measured over a period of 15 h at 37°C. Bacterial survival was calculated by interpolating the OD of a bacterial standard dilution series and the samples and performing a non-linear regression fit analysis. Bar graphs represent bacterial survival in percent for human neutrophils **(A)** or murine neutrophils **(B)**. Data show mean ± SD from four to six independent experiments, **p ≤ 0.001; *p ≤ 0.05; paired, two-tailed students *t*-test.

To address the role of Cdc42 activity in the antibacterial activity of neutrophils, we assessed the *in vitro* killing of *E. coli* and *S. aureus*. Cells treated with the Cdc42 inhibitor were not able to efficiently kill both pathogen species, shown by the increased bacterial survival (Figure 5A). Since this phenomenon could also represent an off-target effect of the Cdc42 inhibitor casin, we also checked the killing efficacy of Cdc42 deficient (Cdc42^Δ/Δ^) bone marrow derived murine neutrophils in comparison to the wild type (Cdc42^fl/fl^) controls. Cdc42^Δ/Δ^ neutrophils also showed a significantly reduced ability to kill *E. coli* and *S. aureus*, represented by the increased bacterial survival (Figure 5B) compared to the Cdc42^fl/fl^ control neutrophils. Since changes in the phagocytosis of both pathogen species might be causative for the changes in killing efficacy, we checked the phagocytosis rate of both pathogens by primary human and bone marrow derived murine neutrophils. There was no change in the phagocytic capacity of human neutrophils with inhibited Cdc42 or murine Cdc42^Δ/Δ^ neutrophils compared to untreated control cells or Cdc42^fl/fl^ cells, respectively (Supplementary Figures 2, 3).

### Inhibition of Cdc42 Activity Results in Phosphorylation Changes of MAPK in Response to fMLP and LPS

**Figure 6:**
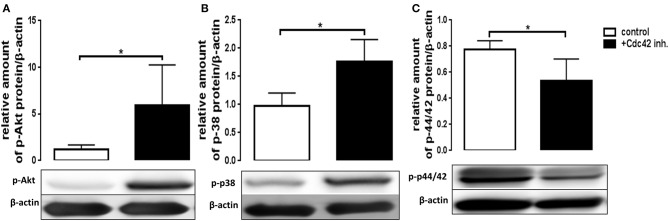
Cdc42 regulates MAPK signaling in fMLP treated neutrophils. Freshly isolated human neutrophils treated with (black bars) or without (white bars) the Cdc42 inhibitor casin (10 μM) for 30 min at 37°C, were stimulated with 1 μM fMLP for 10 min at 37°C. The cells were lysed and analyzed for phosphorylated Akt **(A)**, p38 **(B)**, and p42/44 **(C)** followed by β-actin. Representative blot of three different experiments is shown. Bar graphs represent relative amount of p-p42/44, p-p38, p-Akt normalized by β-actin of three independent experiments. *p ≤ 0.05; paired, two-tailed students *t*-test.

**Figure 7:**
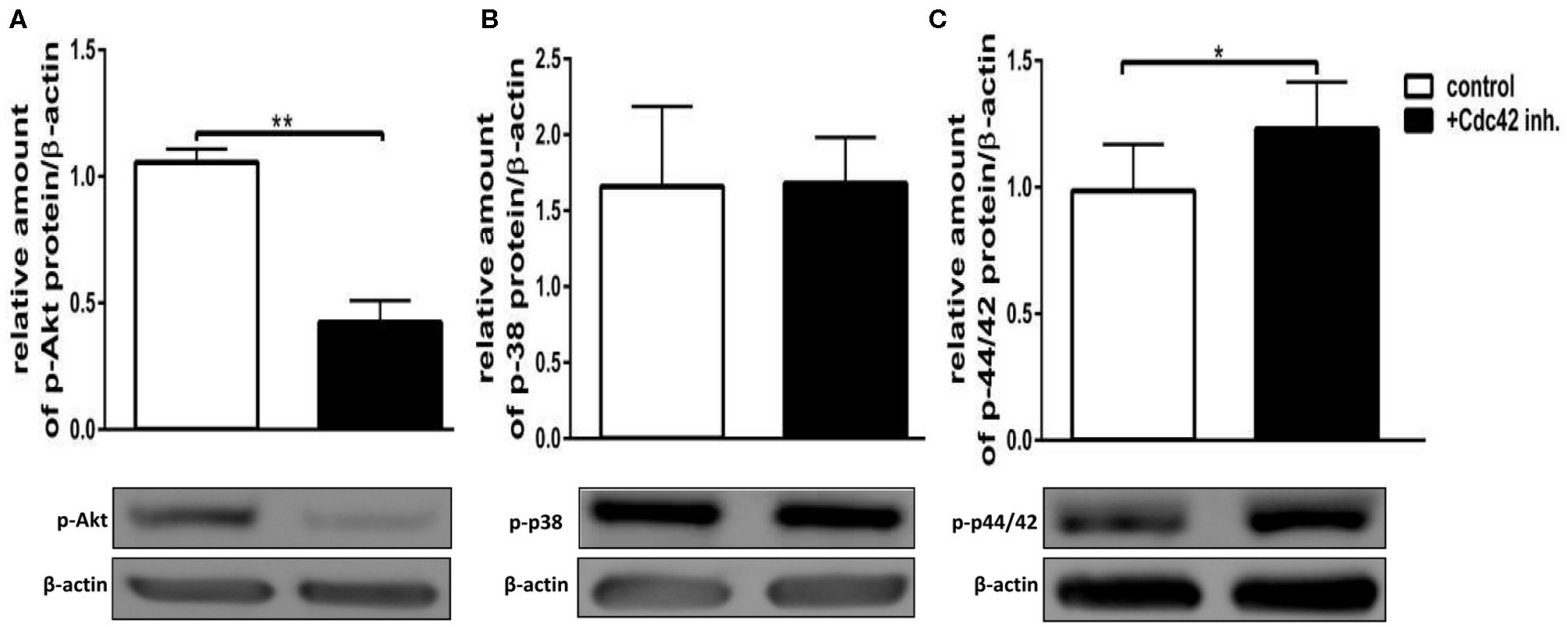
Cdc42 regulates MAPK signaling in LPS treated neutrophils. Freshly isolated human neutrophils treated with (black bars) or without (white bars) the Cdc42 inhibitor casin (10 μM) for 30 min at 37°C, were stimulated with 100 ng/ml LPS for 30 min at 37°C. The cells were lysed and analyzed for phosphorylated Akt **(A)**, p38 **(B)**, and p42/44 **(C)** followed by β-actin. Representative blot of three different experiments is shown. Bar graphs represent relative amount of p-p42/44, p-p38, p-Akt normalized by β-actin of three independent experiments. *p ≤ 0.05; **p ≤ 0.01; paired, two-tailed students *t*-test.

The exact signaling cascade by which Cdc42 controls neutrophil functions is still poorly understood. To get a better understanding of the relevant signaling pathways, we examined the effects of Cdc42 inhibition on the activation of Akt, p38, and p42/44 signaling molecules in response to fMLP and LPS. The mentioned kinases are already known to play different roles in ROS formation, migration, degranulation, and cytokine production of neutrophil granulocytes. Western blot analysis revealed that phosphorylation of p38 and Akt was increased in Cdc42 inhibited cells stimulated with fMLP as compared to fMLP treated control cells (Figures 6A,B). The phosphorylation of p42/44 was reduced in Cdc42 inhibited cells treated with fMLP compared to fMLP only treated cells (Figure 6C). Neutrophils treated with the Cdc42 inhibitor casin and stimulated with LPS showed lower phosphorylation of Akt (Figure 7A) but increased phosphorylation of p44/42 (Figure 7C) compared to LPS only treated cells. No significant change in p38 phosphorylation could be detected (Figure 7B).

## Discussion

Neutrophil granulocytes, as first responders of the innate immune system migrate rapidly toward the site of infection or inflammation. Migration includes multiple steps known to be regulated by RhoGTPases (21, 22). Experiments with Cdc42 deficient neutrophils from transgenic mice showed that this sGTPase is essential for migration by influencing the cell polarity, integrin activation as well as distinct MAPK signaling pathways (22, 23). Another member of the RhoGTPase family, Rac, is also known to be of importance for cell motility and directionality. In addition, Rac is known to play a role in the formation of ROS and in degranulation (17, 24). The fact that both share some GEFs but influence migration differently suggests that they share some signaling pathways while having distinct subsets of downstream targets. This would mean that not only Rac but also Cdc42 plays a special role in controlling several neutrophil effector mechanisms. In the present study we aimed to elucidate the role of Cdc42 in the regulation of functions of primary human neutrophils.

We could show that Cdc42 activity is important for neutrophil motility and directionality in response to various stimulators. These findings confirm data from previous studies showing a migration defect of Cdc42 deficient murine neutrophils (22). Our data suggests that Cdc42 activity is necessary for a proper migration of neutrophil granulocytes in response to chemotactic or chemokinetic stimuli. Hii et al. reported a role of ERK1/2 in random migration and chemotaxis of neutrophils by showing that human neutrophils present lower random migration and chemotaxis in response to fMLP, when ERK1/2 is inhibited (25). We observed here that human neutrophils treated with fMLP and the Cdc42 inhibitor casin show lower levels of phosphorylated p42/44 (ERK1/2), implying that Cdc42 is an upstream regulator of the MAP-kinase ERK1/2. This effect on the phosphorylation of p42/44 is possibly linked to the significantly decreased random and directed locomotion of Cdc42-inhibited neutrophils since the activity of p42/44 appears to be necessary to control downstream targets important for cell migration. As shown in Figure 1, LPS induces a chemokinetic migration of neutrophils rather than chemotactic migration. Inhibition of Cdc42 suppresses the actin-polymerization in neutrophils (7–9), which seems to efficiently block both, the random and directed migration in response to LPS.

The formyl-peptide receptor 1 (FPR1) is expressed by most innate leukocyte subtypes and can be activated upon binding of formyl peptides, like fMLP (26). Once the FPR1 is activated, this receptor is endocytosed and desensitized, which is characterized by the phosphorylation of its intracellular tail by a GPCR kinase (GRK) (26). This receptor internalization reduces the chemotactic migration of neutrophils (26, 27). Similar to fMLP, the complement product C5a is an end-target chemoattractant, and the most potent chemotactic anaphylatoxin that acts through the C5a receptor (C5aR) (27). Subramanian et al. reported, that both C5aR and FPR1 are internalized in response to C5a or fMLP binding, respectively (26). This group used fMLP in a nano-molar concentration and was able to show that FPR1-internalization in dPBL cells can be blocked by using a high concentration of the Cdc42 inhibitor ZCL278 (26). These results were also confirmed by using dPBLs lacking Cdc42, showing the same inhibition of FPR1-internalization in response to fMLP, and additional Cdc42 inhibition (26). In our study, a higher concentration of fMLP was used, and the fMLP-induced chemotaxis was significantly reduced when cells were treated with the Cdc42 inhibitor casin. It is possible that, although Cdc42 inhibition is blocking the FPR1-internalization, the high concentration of fMLP leads to the potentiation of GRK2-function. Subsequently, the increased function of GRK2 might cause the desensitization- and internalization of FPR1, inhibiting chemotaxis of Cdc42-inhibited neutrophils in response to fMLP.

In contrast to fMLP stimulated cells, we showed here an increased chemotactic migration of Cdc42-inhibited neutrophils in response to C5a (Figure 1). Subramanian et al. showed that, similar to FPR1, the C5aR is internalized in response to C5a (26). However, the reported Kd of the C5aR, which is sufficient to induce chemotaxis, was not sufficient to induce receptor internalization (26). A four times higher concentration was necessary to induce the internalization of the C5aR (26). Furthermore, they reported that a lower concentration of C5a induces C5aR-internalization to a much lower extent (26). Similar to the FPR1-internalization, Subramanian et al. showed that Cdc42 inhibition significantly blocks the endocytosis of the C5aR from the neutrophil surface (26). We used in our study a lower C5a concentration then the group of Subramanian, which is a likely reason why the C5aR-internalization cannot be induced. Furthermore, the C5aR-internalization is further blocked by inhibiting Cdc42, as shown by Subramanian et al. (26). Hence, the low concentration of C5a and the addition of the Cdc42 inhibitor are the likely reason for the lack of C5aR-internalization and—desensitization and therefore for increased chemotactic migration of Cdc42-inhibited neutrophils in response to C5a.

Neutrophil granules are classified into primary, secondary, and tertiary granules and secretory vesicles. Primary granules, also known as azurophilic granules are the main storage site for the most toxic mediators, including elastase, cathepsin G, and defensins. Moreover, the enzyme myeloperoxidase (MPO) as well as the cell surface protein CD63 are stored in primary granules (17, 18).

Secretory vesicles, which are formed at the last step of granule differentiation within immature neutrophils contain various important receptors expressed on the surface of neutrophil granulocytes (15, 17). These include Fc-receptors (CD16), LPS- and lipoteichoic acid (LTA) -receptors (CD14), fMLP-receptors as well as complement receptors (CR1). Additionally they contain adhesion molecules like CD11a/b or CD66b and CD18.

Mature, circulating PMNs contain all of the mentioned granules, and upon encountering pathogens or an infection they degranulate. The release of granular contents follows a certain hierarchy, starting with secretory vesicles that represent the biggest predisposition for extracellular release of their content (15, 17). Secretory vesicles are followed by tertiary granules, secondary granules, and finally primary granules (15, 17). This hierarchical mobilization of granules is dependent on calcium and seems to be tightly regulated by different intracellular signaling pathways (15, 17).

Our study showed that the Cdc42 activity is also a regulator of neutrophil granulocyte activation and degranulation, as shown by changes in L-selectin (CD62L) and integrin (CD11b and CD18) expression. The inhibition of Cdc42 resulted in PMN activation shown by increased L-selectin shedding in response to C5a and fMLP. However, inhibition of Cdc42 led to reduced activation of neutrophils in response to LPS. Degranulation of neutrophils, results in enhanced surface expression of CD11b and CD18. We observed that cells treated with the Cdc42 inhibitor casin present significantly increased expression of CD11b and CD18 upon exposure to C5a and fMLP. Inhibition of Cdc42 had an opposing effect on LPS treated neutrophils. Neutrophils treated with the Cdc42 inhibitor casin showed significantly decreased expression of CD11b and CD18 in response to LPS. The opposing effect of Cdc42 inhibition on neutrophils treated with C5a and fMLP on the one hand, and with LPS on the other hand suggests that Cdc42 is both a positive and negative regulator of different signaling pathways.

Neutrophils, upon stimulation, show secretion of most granule types except for primary granules, which fail to undergo exocytosis or show a lower level of expression (28). A previous study presented evidence, that inhibition of actin polymerization was accompanied by an impressive potentiation of degranulation of primary granules (29). A recent study from Mitchell et al. reported a regulatory role of actin remodeling in the primary granule exocytosis by using drugs that inhibit the F-actin polymerization in human neutrophils (28). This group used the surface expression of CD63 and the release of MPO as markers for primary granule exocytosis, and showed that PMNs treated with fMLP show little surface labeling of CD63 (28). However, the inhibition of F-actin polymerization increased the exocytosis of CD63 as well as of MPO, an effect that was attributed by the authors to a prolonged and enhanced activation of Rac1/2 (28). Mitchell et al. concluded from their results that inhibition of F-actin polymerization represents a potent primary granule secretion stimulus that causes sustained Rac activation in neutrophils (28). We showed here a significant upregulation of CD63 expression in unstimulated cells as well as in response to LPS, fMLP and C5a, when Cdc42 was inhibited (Figure 3). Several studies already described that Cdc42 activity regulates the polymerization of F-actin, and that blocking of Cdc42 activity, e.g., with casin, leads to the disruption of F-actin polymerization (7, 8, 21, 22). Consistent with previous data, the stimulation of human neutrophils with LPS, fMLP, or C5a did not significantly increase the release of the primary granule marker CD63. However, upon Cdc42 inhibition, the CD63 surface expression was significantly upregulated. It is to assume that inhibition of Cdc42, and therefore of F-actin polymerization by using casin, is causing a sustained and enhanced activation of the small GTPase Rac. The enhanced activation of Rac could be causative for the increased primary granule exocytosis that was observed in our experiment.

Several kinases are known to play a role in regulating the activation and degranulation of neutrophils. Chen et al. showed that Akt123 activity is important for degranulation, since Akt123 deficient murine PMNs showed significantly lower degranulation upon C5a and fMLP stimulation (30). Furthermore, Rizoli et al. reported that p38 activity is important for the L-selectin shedding by showing that inhibition of p38 reduced the L-selectin-shedding in response to fMLP in human neutrophils (31). We showed here that Cdc42 inhibited human neutrophils stimulated with fMLP display increased phosphorylation of Akt and p38. In contrast, Cdc42 inhibited neutrophils stimulated with LPS showed decreased phosphorylation of Akt. These results suggest that the activation- and degranulation changes in response to fMLP or LPS can be in part regulated by Akt and p38. The involvement of p38 is possibly linked to a role of this kinase in the activation or membrane insertion (exocytosis) of the L-selectin-sheddase (31). Alternatively, p38 could also be important to render L-selectin susceptible for proteolytic cleavage, causing an increased L-selectin shedding due to high p38 activity.

The formation of ROS is one of the hallmarks associated with pro-inflammatory and antimicrobial actions of neutrophil granulocytes (24). The NADPH-oxidase as one of the major producers of ROS and its catalytic core is localized at cellular membranes including those of intracellularly stored granules (24). Degranulation and ROS formation display closely related processes (24). We showed in this study that cells treated with casin produce significantly more ROS in response to C5a and fMLP but decreased ROS in response to LPS. These findings correlate with the degranulation data, and imply that the increased degranulation in response to C5a and fMLP are connected to the enhanced formation of intracellular ROS. Shared signaling events between degranulation and ROS formation can be causative for this observation. Chen et al. reported that Akt deficient murine neutrophils show significantly less ROS formation in response to C5a and fMLP. This indicates that Akt-activity in part regulates ROS formation. Indeed we showed that Cdc42-inhibited cells present a higher Akt123 phosphorylation when stimulated with fMLP but a lower Akt phosphorylation when stimulated with LPS.

The ultimate goal of all neutrophil granulocyte effector mechanisms is the killing of pathogens. All effector mechanisms work in concert to effectively eliminate bacteria or other pathogens. We could show that Cdc42 activity is necessary for neutrophil granulocytes to efficiently kill *E. coli* and *S. aureus in vitro*. Both species showed significantly increased survival in Cdc42-inhibited human and in Cdc42 deficient murine neutrophils. This data indicates that Cdc42 is a major regulator of neutrophil antimicrobial activity to efficiently clear pathogens. Gram positive and gram-negative bacteria are recognized by TLR2 and TLR4, respectively (32). Commercially available LPS contain significant amounts of bacterial lipopeptides, and can therefore be seen as dual TLR2 and TLR4 agonists (33). We observed a significant reduction of LPS-induced neutrophil activation, degranulation, and ROS production upon Cdc42 inhibition. This observation is most likely connected to the reduced ability of Cdc42-inhibited or—deficient neutrophils to efficiently kill *E. coli* and *S. aureus*.

In summary, we showed here that the sGTPase Cdc42 activity plays a role in neutrophil granulocytes beyond controlling their migration. Cdc42 is a major regulator of ROS formation, degranulation as well as activation of neutrophils in a stimulus dependent manner. We reported that Cdc42 seems to be a positive regulator of LPS induced effector mechanisms while it appears to be a negative regulator of GPCR-triggered neutrophil functions. Importantly, Cdc42 activity was found to be required for effective killing of microbial pathogens by both human and murine neutrophils.

## Data Availability Statement

The raw data supporting the conclusions of this article will be made available by the authors, without undue reservation.

## Ethics Statement

The studies involving human participants were reviewed and approved by Ethics Committee of the University of Lübeck (18-187). The patients/participants provided their written informed consent to participate in this study. The animal study was reviewed and approved by Animal Care Ethics Committee of Cincinnati Children's Hospital Medical Center.

## Author Contributions

HT designed and performed experiments, analyzed data, and wrote the manuscript. SM assisted to perform experiments. M-DF and TL supported to design experiments, interpreted data and revised the manuscript.

## Conflict of Interest

The authors declare that the research was conducted in the absence of any commercial or financial relationships that could be construed as a potential conflict of interest.
