# Supplementary material for: The Small GTPase Cdc42 Is a Major Regulator of Neutrophil Effector Functions
Source: Front Immunol. 2020 Jun 12;11:1197. doi: 10.3389/fimmu.2020.01197 (PMC7304460; doi:10.3389/fimmu.2020.01197)
Supplement: Supplementary file 1 [file Data_Sheet_1.pdf]

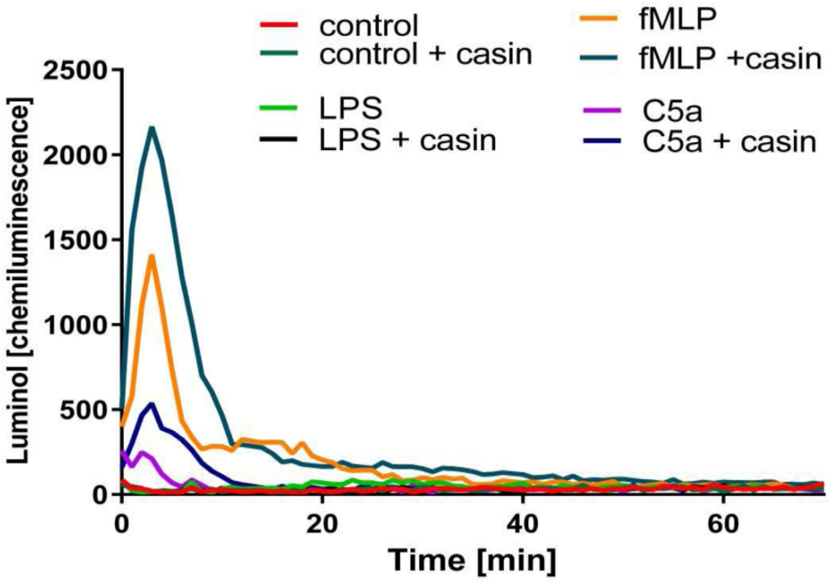

Suppl. Figure 1: Effect of Cdc42 inhibition on ROS production

Primary human neutrophils were incubated with or without 10 µM casin. Subsequently, luminol was added and the ROS production of **unstimulated cells** or in response to **100 ng/ml LPS, 10 nM C5a or 1 µM fMLP** was measured. The release of H_2O_2 (lumiol chemiluminescence) was detected for 1 hr at 37 °C. Line graph of one representative experiment showing the luminol chemiluminescence over time.

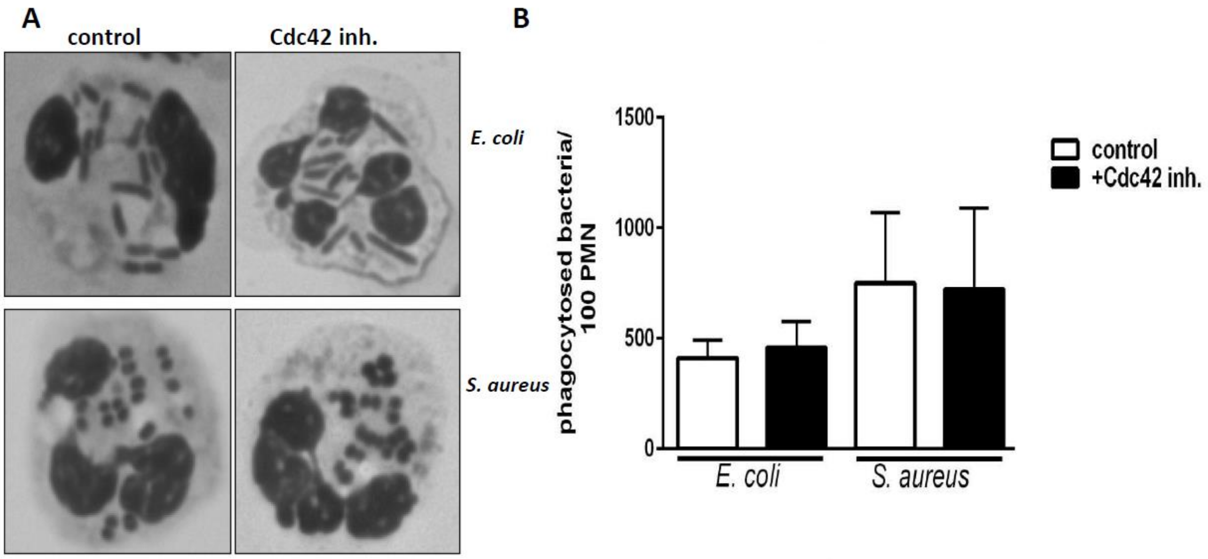

Suppl. Figure 2: Inhibition of Cdc42 does not change the phagocytosis of *E. coli* or *S. aureus* by human neutrophils

Freshly isolated human neutrophils, treated with or without 10 µM casin were incubated with *Escherichia coli* or *Staphylococcus aureus* for 30 min at 37 °C and subsequently cytocentrifuged. **(A)** Representative microscopy images of cells showing phagocytosed *E. coli* and *S. aureus.* **(B)** Bar graphs

representing the number of phagocytosed *E. coli* or *S. aureus* by 100 untreated (white bars) or casin treated cells (black bars). Data show mean ± SD from four independent experiments.

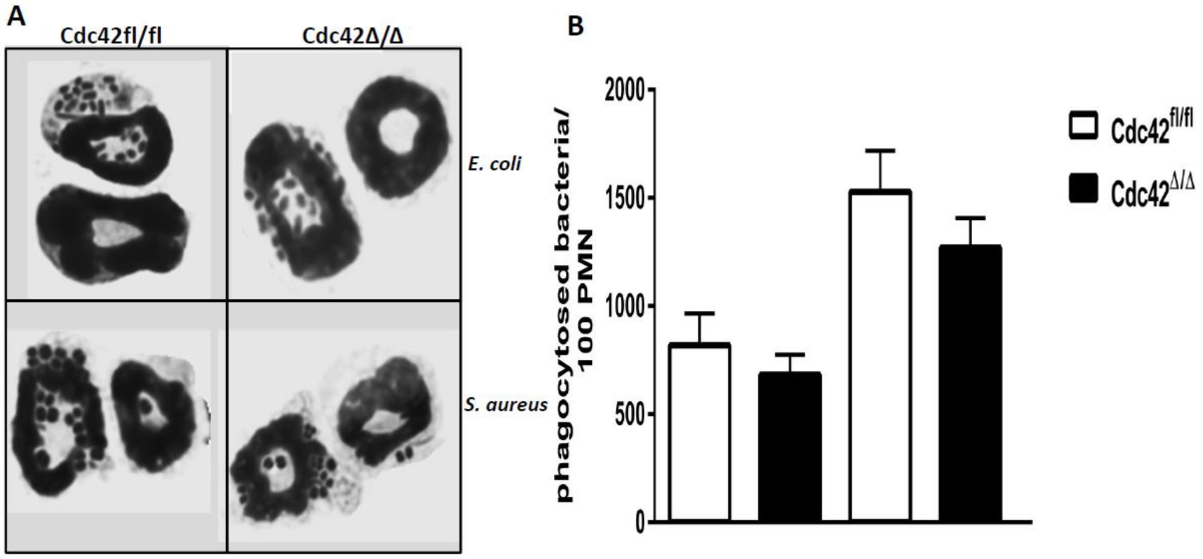

Suppl. Figure 3: The absence of Cdc42 does not change the phagocytosis of *E. coli* or *S. aureus* by murine neutrophils

Freshly isolated bone marrow derived murine neutrophils from $Cdc42^{fl/fl}$ and $Cdc42^{\Delta/\Delta}$ mice were incubated with *Escherichia coli* or *Staphylococcus aureus* for 30 min at 37 °C and, subsequently, cytocentrifuged. **(A)** Representative microscopy images of $Cdc42^{fl/fl}$ and $Cdc42^{\Delta/\Delta}$ cells showing phagocytosed *E. coli* and *S. aureus.* **(B)** Bar graphs represent the number of phagocytosed *E. coli* or *S. aureus* by 100 $Cdc42^{fl/fl}$ (white bars) or $Cdc42^{\Delta/\Delta}$ cells (black bars). Data show mean ± SD from four independent experiments.
